# Continuous Suture Technique in Surgical Aortic Valve Replacement: Early and Mid-Term Outcomes in a Real-World Cohort Including Combined Procedures

**DOI:** 10.3390/jcdd13060277

**Published:** 2026-06-19

**Authors:** Eray Aksoy, Zumrut Tuba Demirozu, Sami Gurkahraman, Mehmet Sanser Ates

**Affiliations:** 1 Department of Cardiovascular Surgery, Vehbi Koc Foundation American Hospital, 34365 Istanbul, Türkiye; samig@amerikanhastanesi.org (S.G.); sanserates@amerikanhastanesi.org (M.S.A.); 2 Department of Cardiovascular Surgery, Koc University Faculty of Medicine, 34010 Istanbul, Türkiye; zdemirozu@kuh.ku.edu.tr

**Keywords:** surgical aortic valve replacement, continuous suture technique, aortic valve disease, prosthetic valve, paravalvular leak, cardiac surgery, mid-term outcomes

## Abstract

The continuous suture technique is not routinely used in surgical aortic valve replacement (SAVR), and data regarding its clinical outcomes remain limited. This retrospective observational study evaluated early and mid-term outcomes after continuous suture SAVR in a real-world cohort. Eighty-eight consecutive patients who underwent SAVR using a continuous suture technique between November 2015 and July 2024 were included. Both isolated and concomitant procedures were analyzed. The operative technique consisted of three 2-0 polypropylene sutures, one placed along each aortic cusp. Clinical outcomes, postoperative complications, and survival were assessed. The mean age was 62.22 ± 15.22 years, and 71.6% of patients were male. Bioprosthetic valves were implanted in 61.4% of cases, and the mean prosthesis size was 25.02 ± 0.93 mm. Concomitant procedures were performed in 86.4% of patients. There were no in-hospital deaths. New-onset atrial fibrillation occurred in 26.1% of patients, and permanent pacemaker implantation was required in 3.4%. The median cross-clamp time was 41.50 min. During a mean follow-up of 18.38 months, one- and three-year overall survival was 92.9%. No prosthetic valve dysfunction related to thrombus, pannus formation, or clinically significant paravalvular leak was observed. Continuous suture SAVR appears feasible and safe, with acceptable early and mid-term outcomes, although the retrospective, non-comparative design requires cautious interpretation.

## 1. Introduction

Surgical aortic valve replacement (SAVR) remains the standard treatment for aortic valve disease, with well-established long-term outcomes despite increasing patient complexity [1,2]. In addition to patient-related factors, technical aspects of valve implantation play an important role in postoperative hemodynamic performance and complication rates. The conventional interrupted suture technique is widely used because of its reproducibility and perceived safety. However, it has been associated with several limitations, including patient–prosthesis mismatch (PPM) and prosthesis-related complications such as thrombosis or pannus formation [3,4,5], and may also be associated with paravalvular leak in certain settings.

The continuous suture technique has been used as an alternative approach and may offer several practical advantages. Previous studies have described this technique as simple, rapid, and secure, with the potential to reduce cross-clamp and cardiopulmonary bypass times [6,7]. In addition, by minimizing the number of suture knots and avoiding pledgeted material, continuous suturing may facilitate implantation of larger prostheses and contribute to improved hemodynamic performance [8].

Despite these potential advantages, concerns regarding atrioventricular conduction disturbances, increased pacemaker requirement, and paravalvular leak have limited its widespread use [9]. Available data remain limited and, in some aspects, inconsistent, particularly in heterogeneous patient populations undergoing combined procedures. In our study, we aimed to evaluate the early and mid-term outcomes of consecutive patients undergoing isolated and combined SAVR using a continuous suture technique in a real-world setting.

## 2. Materials and Methods

**Figure 1:**
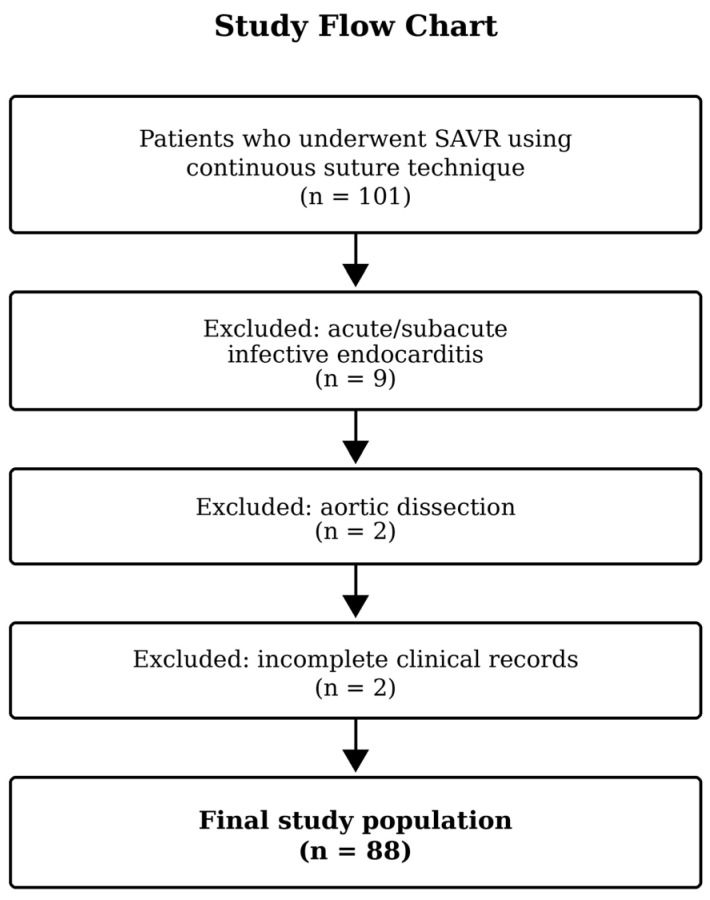
Study flow chart.

This retrospective observational cohort study included patients aged ≥ 14 years who underwent open surgical aortic valve replacement (SAVR) using a continuous suture technique at a tertiary care center between November 2015 and July 2024. A total of 101 patients underwent SAVR during the study period. Patients with acute or subacute infective endocarditis (n = 9), aortic dissection (n = 2), and incomplete clinical records (n = 2) were excluded. The final study population consisted of 88 patients. The study flow charts as shown in Figure 1. The study population was heterogeneous and included both isolated and combined SAVR procedures, as well as patients receiving mechanical and bioprosthetic valves, reflecting routine surgical practice. The study was approved by the Koc University Committee on Human Research, and the requirement for individual informed consent was waived due to the retrospective design.

The implanted prostheses included St. Jude Medical Epic or Epic Plus stented tissue valves for bioprosthetic valve replacement (Abbott, Santa Clara, CA, USA) and ATS mechanical heart valves for mechanical valve replacement (Medtronic, Minneapolis, MN, USA). Combined SAVR was defined as SAVR performed together with any additional cardiac procedure.

Demographic characteristics, comorbidities, and previous cardiac surgical history were obtained from institutional digital records. Baseline echocardiographic parameters, including left ventricular ejection fraction, interventricular septal thickness, left ventricular end-diastolic diameter, systolic pulmonary artery pressure, and transaortic gradient and velocity (in patients with isolated aortic stenosis), were recorded. Operative data included procedure type (isolated or combined SAVR), concomitant procedures, prosthetic valve type and size, reoperation status, root enlargement, and aortic cross-clamp time.

The primary outcome was cardiac-related mortality. Secondary outcomes included in-hospital and follow-up clinical outcomes. In-hospital outcomes included mortality, new-onset atrial fibrillation, sinus bradycardia, atrioventricular conduction disturbances, need for permanent pacemaker implantation, respiratory complications (atelectasis or pulmonary consolidation, re-intubation), reoperation for bleeding or cardiac tamponade, renal dysfunction (serum creatinine > 1.5 mg/dL), infection, and cerebrovascular events. Postoperative rhythm monitoring was performed using 24-h telemetry in all patients during the hospital stay.

### 2.1. Surgical Technique

**Figure 2:**
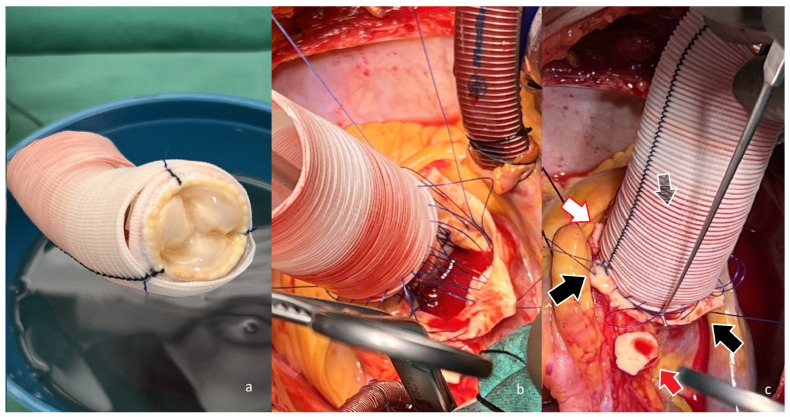
Continuous suture technique in Bentall procedure. (**a**) A composite conduit was prepared by suturing a 27-mm Abbott Epic Plus bioprosthetic valve to a 32-mm Gelweave vascular graft. In a 56-year-old female patient (body mass index 25.5 kg/m^2^), the largest feasible bioprosthetic valve was preferred due to refusal of long-term anticoagulation therapy and the anticipated feasibility of future valve-in-valve transcatheter aortic valve replacement. (**b**) Continuous suture line initiated at the non-coronary–left coronary commissure and advanced in a clockwise direction from the surgeon’s perspective; the suture line is completed prior to seating the prosthesis using the parachute technique. (**c**) The prosthetic valve is seated within the aortic annulus and secured using a blunt hook. Black arrows indicate the three commissural suture junctions; the junction between the non-coronary and right coronary cusps is obscured behind the graft (gray arrow). The white arrow indicates the right coronary button, and the red arrow indicates the left coronary button prepared for reimplantation.

All operations were performed by the same surgical team under general anesthesia. Following a full median sternotomy, cardiopulmonary bypass was established via distal aortic arch cannulation and either right atrial two-stage or bicaval venous cannulation. After application of the aortic cross-clamp, cardiac arrest was achieved with antegrade crystalloid cardioplegia, and myocardial protection was maintained with intermittent retrograde cardioplegia throughout the procedure. An oblique aortotomy was performed 0.5–1.0 cm above the sinotubular junction and extended toward the non-coronary sinus. In cases where the aortic root was too small to accommodate an appropriately sized prosthesis, aortic root enlargement was performed by extending the aortotomy below the non-coronary cusp and augmenting the defect with an eye-drop-shaped Dacron patch. A continuous suture technique was routinely used for valve implantation. Three 2-0 polypropylene sutures were placed, one for each aortic cusp, and tied at the commissures. The first stitch was placed at the non-coronary–left coronary commissure, passed from the ventricular to the aortic side, and secured. The suture was then run clockwise along the annulus and counterclockwise along the sewing cuff. Standard-depth bites were used along the non-coronary and left coronary cusps, whereas shallower bites were taken along the right coronary cusp to minimize the risk of conduction system injury. After sequential tightening of the sutures, the prosthetic valve was seated, and the aortotomy was closed after confirming adequate valve motion. The procedure was completed in the standard fashion, and two temporary epicardial pacing wires were placed in all patients. In patients undergoing a Bentall procedure, the largest feasible mechanical or bioprosthetic valve was sutured to a Gelweave vascular graft to create a composite conduit (Figure 2a). The conduit was then implanted into the aortic annulus using the same continuous technique with three 2-0 polypropylene sutures (Figure 2b,c). Coronary ostia were prepared as buttons and reimplanted into the graft using 6-0 polypropylene sutures.

### 2.2. In Hospital Progress and Follow-Up

Patients who were hemodynamically stable and did not have respiratory failure were extubated in the operating room and transferred to the intensive care unit breathing spontaneously. Chest drains were removed on the first postoperative day, after which patients were transferred to the ward. For patients requiring temporary pacing, intrinsic rhythm was assessed at regular intervals, and pacing was continued in the presence of persistent bradyarrhythmia or advanced atrioventricular block. If pacing dependence persisted beyond seven days, cardiology was consulted for permanent pacemaker evaluation. Warfarin therapy started on the first postoperative day; it was discontinued after three months in patients with bioprosthetic valves and continued lifelong in those with mechanical valves. Prior to discharge, an INR between 2.5 and 3.5 was ensured.

Follow-up data were obtained from hospital electronic records and routine outpatient clinic visits. Survival status and causes of death were determined based on hospital records and available clinical documentation. For patients who were alive at last follow-up, the most recent clinical visit was used to confirm survival status. Patients were censored at the time of last known follow-up. Echocardiographic follow-up was performed as part of routine clinical care, and available reports were reviewed for prosthetic valve function. Prosthetic valve-related complications, including thrombosis, pannus formation, and paravalvular leak, were assessed based on echocardiographic reports and clinical records.

Generative artificial intelligence (GAI) tools were used only to assist with language editing, academic phrasing, and formatting of the manuscript. No GenAI tool was used for study design, data collection, statistical analysis, data interpretation, image or figure generation, or the formulation of scientific conclusions. All content was reviewed, verified, and approved by the authors, who take full responsibility for the final version of the manuscript.

### 2.3. Statistical Analysis

Statistical analyses were performed using IBM SPSS Statistics for Windows, version 21.0 (IBM Corp., Armonk, NY, USA). Continuous variables were first tested for normality using the Shapiro–Wilk test. Variables with normal distribution are presented as mean ± standard deviation, whereas non-normally distributed variables are presented as median and interquartile range. Categorical variables are expressed as counts and percentages. Survival and rehospitalization-free survival were evaluated using the Kaplan–Meier method. A *p* value <0.05 was considered statistically significant.

## 3. Results

The mean age of the study population was 62.22 ± 15.22 years, and 63 patients (71.6%) were male. The indications for aortic valve replacement included concomitant aortic regurgitation and stenosis in 33 patients (37.5%), isolated aortic stenosis in 23 patients (26.1%), and isolated aortic regurgitation in 32 patients (36.4%). Twenty-two patients (25.0%) had a bicuspid aortic valve (Table 1).

Bioprosthetic valves were used in 54 patients (61.4%), while the remaining patients received mechanical prostheses. The mean prosthesis size was 25.02 ± 0.93 mm. Valve sizes were 23 mm in 9 patients (10.2%), 25 mm in 69 patients (78.4%), and 27 mm in 10 patients (11.4%) (Table 2). Aortic valve replacement was a reoperation in 6 patients (6.8%). Six patients had a history of prior valve surgery, and one patient had previously undergone coronary artery bypass grafting.

Regarding operative characteristics, 12 patients (13.6%) underwent isolated SAVR, whereas 76 patients (86.4%) underwent combined procedures. Among these, aortic conduit replacement was performed in 47 patients (53.4%), coronary artery bypass grafting in 36 patients (40.9%), and concomitant mitral valve surgery in 18 patients (20.5%). Aortic root enlargement was required in 9 patients (10.2%), and 6 patients (6.8%) underwent reoperative surgery.

The median aortic cross-clamp time for the overall cohort was 41.50 min (IQR, 34.00–52.00). In isolated SAVR cases (n = 12), the median cross-clamp time was 28.50 min (IQR, 26.00–31.00), whereas in combined procedures (n = 76), it was 44.00 min (IQR, 38.00–54.75). Within the combined procedures, median cross-clamp times were 41.00 min (IQR, 38.00–50.00) for aortic conduit replacement (n = 47), 46.00 min (IQR, 39.25–58.50) for coronary artery bypass grafting (n = 36), and 54.50 min (IQR, 44.00–70.50) for concomitant mitral valve surgery (n = 18). Cross-clamp times were 52.00 min (IQR, 41.00–71.50) in patients undergoing aortic root enlargement (n = 9) and 51.50 min (IQR, 36.25–70.50) in reoperative cases (n = 6).

There were no in-hospital deaths. New-onset atrial fibrillation occurred in 23 patients (26.1%). Sinus rhythm was restored in 21 patients after medical cardioversion and in 2 patients after direct current cardioversion. Sinus bradycardia without conduction delay was observed in 12 patients (11.5%). Three patients (3.4%) developed third-degree atrioventricular block and underwent permanent pacemaker implantation before discharge (Table 3).

### Follow-Up and Survival

The mean follow-up duration was 18.38 months (median, 13.80 months; IQR, 2.73–24.40 months). During follow-up, four patients died, none due to cardiac causes. One patient died from chronic obstructive pulmonary disease, two from intracerebral hemorrhage, and one from pulmonary adenocarcinoma. The mean estimated overall survival was 75.62 months (95% CI, 70.64–80.59 months), with one- and three-year survival rates of 92.9%. Cardiac- or surgery-related rehospitalization occurred in 14 patients (15.9%). The causes of rehospitalization included acute-onset atrial fibrillation in 7 patients, pleural effusion requiring drainage in 4 patients, atrioventricular block requiring permanent pacemaker implantation in 1 patient, transient ischemic attack in 1 patient, and revision for wound dehiscence in 1 patient. The mean estimated cardiac- or surgery-related rehospitalization-free survival was 56.64 months (95% CI, 46.65–66.64 months). One- and three-year rehospitalization-free survival rates were 80.0% and 63.3%, respectively. During follow-up, no cases of prosthetic valve dysfunction due to thrombus, pannus formation, or paravalvular leak were observed.

## 4. Discussion

In this study, we evaluated the early and mid-term outcomes of surgical aortic valve replacement using a continuous suture technique in our center, including both isolated and combined procedures. The study population was heterogeneous, reflecting routine clinical practice. Our aim was to assess the feasibility and clinical outcomes of this technique when used in a real-world setting, given that available data in the literature remain limited, particularly in more complex surgical cases.

The continuous suture technique differs from the conventional interrupted approach mainly by reducing the number of suture knots and avoiding pledgeted material. Pledget-reinforced sutures have been suggested to affect local hemodynamics and prosthesis–tissue interaction; however, their impact on thrombus formation and pannus development is not clearly defined [5]. Reducing the amount of foreign material at the annular level may be advantageous, although its clinical relevance remains uncertain.

Because this technique differs from conventional practice and raises concerns regarding potential injury to the atrioventricular conduction system, its use has historically been limited, and data on long-term outcomes remain relatively scarce. Earlier studies reported higher rates of permanent pacemaker implantation in patients undergoing continuous suture SAVR, with incidences up to 17.5% [10]. More recent studies, however, have reported acceptable pacemaker rates and no clear increase in conduction disturbances when the technique is applied appropriately. Sultan et al. [11], in a propensity-matched analysis, reported comparable outcomes between continuous and interrupted suturing techniques, with low permanent pacemaker incidence in both groups. Similarly, Niclauss et al. [12] reported a low incidence of permanent pacemaker implantation following continuous suturing, particularly in patients without preexisting conduction disorders or infective endocarditis. In our series, permanent pacemaker implantation was required in 3 patients (3.4%), all of whom underwent combined procedures. These findings suggest that the risk of conduction disturbances requiring pacemaker implantation remains low and may be influenced more by procedural complexity than by the suturing technique itself.

Sutureless aortic valve prostheses have gained increasing popularity, primarily due to their ability to reduce cross-clamp and cardiopulmonary bypass times and to facilitate technical simplicity during valve implantation [13]. Contemporary evidence suggests that sutureless valves have been associated with shorter operative times, particularly in complex or combined procedures. A recent network meta-analysis comparing transcatheter aortic valve replacement (TAVR), sutureless SAVR, and conventional surgical techniques demonstrated that open surgical approaches remain advantageous in terms of paravalvular leak rates, whereas minimally invasive techniques offer procedural benefits [14]. However, sutureless valves have also been associated with certain drawbacks, including higher rates of conduction disturbances and permanent pacemaker implantation in some series. In this context, continuous suture SAVR may represent a balanced alternative, combining the procedural familiarity of conventional surgery with potential reductions in operative complexity. In our cohort, cross-clamp times remained within a relatively low range despite the high proportion of combined procedures, supporting the practical efficiency of this technique in real-world surgical settings.

Concerns regarding an increased risk of paravalvular leak with continuous suture SAVR have been reported in earlier studies [15], although the reported rates vary and appear to be influenced by surgical technique. Choi et al. [7] reported no cases of clinically significant periprosthetic leak in their early experience with continuous suturing, suggesting that careful annular preparation and appropriate suture technique may reduce this risk. Similarly, technical factors such as suture material and the depth of annular bites may affect valve seating and sealing. In our series, no patient developed more than trivial paravalvular leak during follow-up, despite the absence of additional reinforcing material such as pericardial strips. Unlike many conventional approaches, we routinely used three separate polypropylene 2-0 sutures rather than a single continuous suture, similar to the technique described by Stamou et al. [6]. This approach may improve prosthetic valve stability and provide more uniform annular fixation. Overall, our findings indicate that, when performed with careful technique, continuous suturing can provide reliable valve seating without an increased risk of clinically significant paravalvular leak.

Patient–prosthesis mismatch (PPM) is a well-known issue in patients undergoing aortic valve replacement and is associated with suboptimal hemodynamic performance, including higher transvalvular gradients and delayed left ventricular mass regression [16]. In surgical aortic valve replacement, predicting PPM remains challenging, as prosthesis sizing is determined intraoperatively, unlike transcatheter approaches where preprocedural imaging allows more precise annular assessment. The indexed effective orifice area (iEOA), calculated by relating prosthetic valve area to body surface area, has been used as a surrogate parameter; however, its clinical applicability and threshold values remain debated, particularly in heterogeneous patient populations and across different body habitus groups [6,17]. Previous studies have shown that a considerable proportion of patients undergoing SAVR receive relatively small prostheses, with moderate PPM reported in up to 60% of cases in some series and severe PPM in approximately 5–10% [18,19]. These observations have increased the focus on strategies aimed at implanting the largest feasible prosthesis, including aortic root enlargement when necessary, despite the associated increase in operative complexity [20,21]. In our series, a prosthesis size of 25 mm or greater was used in most patients, while root enlargement was required in only a small proportion, suggesting that adequate valve sizing can be achieved in many cases. In our practice, the continuous-suture technique was used as the routine implantation approach in eligible SAVR cases rather than being reserved exclusively for patients with a small aortic annulus. However, in patients with a relatively small annulus, the reduced amount of annular foreign material and absence of pledgets may facilitate implantation of an adequately sized prosthesis. Nevertheless, because PPM was not formally assessed using indexed effective orifice area criteria, this potential advantage should be interpreted as hypothesis-generating rather than definitive. Within these limitations, the distribution of prosthesis sizes in our cohort suggests that continuous suturing may facilitate implantation of appropriately sized valves without routinely requiring root enlargement, even in a population with a high proportion of combined procedures.

This study has several limitations. First, the absence of a control group using the interrupted suture technique limits direct comparison regarding the safety and effectiveness of the continuous suture approach. In addition, the retrospective design and the fact that all procedures were performed at a single center by the same surgical team may limit the generalizability of the findings. The study population was also heterogeneous, including both isolated and combined procedures, which may have influenced the observed outcomes. Although follow-up duration was reported, survival status and clinical outcomes were based on available hospital records and outpatient follow-up data, and longer-term outcomes may not have been fully captured. Finally, the relatively small sample size and limited follow-up period do not allow firm conclusions regarding long-term results.

## 5. Conclusions

In conclusion, in our experience, the continuous suture technique provided acceptable early and mid-term outcomes in a heterogeneous patient population undergoing surgical aortic valve replacement. The technique allowed implantation of adequately sized prosthetic valves while maintaining relatively short cross-clamp times, even in combined procedures. However, given the retrospective design and the absence of a comparative control group, our findings should be interpreted with caution. Further studies, particularly prospective analyses, are required to better define the role of this technique in routine clinical practice.

## Figures and Tables

**Table 1:** Baseline data.

Variable	Mean ± SD	n (%)
Age	62.22 ±15.22	
Males		63 (71.6%)
Body mass index (kg/m^2^)	28.93 ± 5.33	
Diabetes		27 (30.7%)
Hypertension		50 (56.8%)
Dyslipidemia		14 (15.9%)
Chronic pulmonary disease		11 (12.5%)
Coronary artery disease		31 (35.2%)
Chronic renal failure		4 (4.5%)
History of cerebrovascular disease		7 (8.0%)
Ascending aortic aneurysm		36 (40.9%)
Peripheral artery disease		4 (4.5%)
Previous CABG		1 (1.1%)
Previous valve surgery		6 (6.8%)
Previous other cardiac surgery		2 (2.3%)
Baseline echocardiography		
Aortic regurgitation and stenosis		33 (37.5%)
Isolated aortic stenosis		23 (26.1%)
Isolated aortic regurgitation		32 (36.4%)
Bicuspid aortic valve		22 (25.0%)
Left ventricle ejection fraction (%)	55.84 ± 9.14	
Interventricular septum (cm)	1.19 ± 0.19	
LVEDD (cm)	5.20 ± 0.67	
Systolic pulmonary artery pressure	32.68 ± 10.26	
Mean transaortic gradient *	50.65 ± 22.12	
Mean transaortic velocity *	4.18 ± 1.33	

* In patients with isolated aortic stenosis (n = 23).

**Table 2:** Operative Parameters.

Variable	n (%)	Valve Size		
		23 mm	25 mm	27 mm
Overall	88 (100%)	9 (10.2%)	69 (78.4%)	10 (11.4%)
Isolated SAVR	12 (13.6%)	2	9	1
Combined	76 (86.4%)	7 (9.2%)	60 (78.9%)	9 (11.8%)
Aortic Conduit	47 (53.4%)	2 (4.3%)	37 (78.7%)	8 (17.0%)
CABG	36 (40.9%)	2 (5.6%)	31 (86.1%)	3 (8.3%)
Mitral valve surgery	18 (20.5%)	5 (27.8%)	13 (72.7%)	0
Root enlargement	9 (10.2)	3	6	0
Reoperation	6 (6.8%)	2	4	0

**Table 3:** Post-operative clinical morbidities and echocardiographic findings.

Variable	n (%)
Clinical morbidities	
Atrial fibrillation	23 (26.1%)
Lung atelectasis/consolidation	16 (18.2%)
Repeat intubation/mechanic ventilation	2 (2.3%)
Revision for bleeding/tamponade	6 (6.8%)
Elevated serum creatinine (>1.5 mg/dL)	3 (3.4%)
Infection	4 (4.5%)
Cerebrovascular event	2 (2.3%)
Permanent pacemaker	3 (3.4%)
Echocardiography	
Ejection fraction (%)	55.77 ± 7.39
Transvalvular gradient of the aortic valve prosthesis	11.56 ± 4.72
Transvalvular velocity of the aortic valve prosthesis	2.14 ± 0.50
In-hospital death	0

## Data Availability

The data supporting the findings of this study are not publicly available due to privacy and ethical restrictions related to patient confidentiality. De-identified data may be made available from the corresponding author upon reasonable request and subject to institutional and ethical approval.

## Abbreviations

The following abbreviations are used in this manuscript:

CABG	Coronary artery bypass grafting
LVEDD	Left ventricle end diastolic diameter
PPM	Patient–prosthesis mismatch
SAVR	Surgical aortic valve replacement
